# An Innovative Physical Therapy Intervention for Chronic Pain Management and Opioid Reduction Among People Living with HIV

**DOI:** 10.1089/biores.2020.0006

**Published:** 2020-12-08

**Authors:** Sara D. Pullen, Carlos del Rio, Daniel Brandon, Ann Colonna, Meredith Denton, Matthew Ina, Grace Lancaster, Anne-Grace Schmidtke, Vincent C. Marconi

**Affiliations:** ^1^ Division of Physical Therapy, Department of Rehabilitation Medicine, Emory University School of Medicine, Atlanta, Georgia.; ^2^ Division of Infectious Disease, Department of Medicine, Emory University School of Medicine, Atlanta, Georgia.; ^3^ Department of Global Health, Emory University Rollins School of Public Health, Atlanta, Georgia.

**Keywords:** chronic pain, opioids, physical therapy

## Abstract

Chronic pain management has become a treatment priority for people living with HIV (PLH), and PLH may be at increased risk for opioid addiction. Physical therapy (PT) has been shown to be effective as a nonpharmacological method of chronic pain management; however, there is a gap in research examining the role of PT for chronic pain, especially as it relates to opioid reduction, in this patient population. This retrospective study evaluated pain level and opioid use before and after PT intervention among HIV-positive adults with chronic pain on chronic opioid therapy (*n* = 22). The study was conducted at a multidisciplinary AIDS clinic in Atlanta, GA. Outcome measures were self-reported pain on the numerical rating scale (0–10) and morphine milligram equivalents (MMEs), which measure opioid use. A majority of patients (77%) demonstrated a decrease in pain by the conclusion of the study period; however, only 18.2% of patients reported decreased pain as well as a decrease in MMEs. The most common PT treatments used among the patients with a decrease in pain and/or opioid use included home exercise programs, manual therapy, and self-pain management education. Eighty percent of the participants who did not decrease opioid use reported a decrease or elimination of pain by the end of the PT intervention. This reflects the need for careful consideration of the complexity of opioid use and addiction, and the importance of a multidisciplinary team to best serve the needs of PLH aiming to decrease chronic pain and opioid use.

## Introduction

Approximately 20% of adults suffer from chronic pain in the United States, costing an estimated $635 billion in both direct and indirect costs.^1,2^ In their 2017 report *Clinical Practice Guideline for the Management of Chronic Pain in Patients Living With HIV,* the HIV Medical Association stated that current estimates of chronic pain range from 39% to 85% among people living with HIV (PLH), as compared with 11% in the general population.^3^ The causes of chronic pain in this patient population are multifactorial, with etiologies ranging from direct viral infection, medication side effects, underlying medical condition, inflammation, past physical and emotional trauma, or an unknown cause.^3^

There is a complex history of opioid use disorder, substance abuse history, and pain management in the United States. The standard of care for chronic pain has historically been a pharmacological approach that heavily included the use of opioid analgesics.^4–6^ Recent federal guidelines have outlined recommendations for pain mitigation in the context of the ongoing opioid epidemic. The Centers for Disease Control and Prevention (CDC)'s *Guideline for Prescribing Opioids for Chronic Pain* reports that there is no evidence to support long-term benefit of long-term opioids use for chronic pain; there are potential serious harms of opioids, including opioid use disorder, overdose, and accidental injury; and that extensive evidence points to both the increased benefits and decreased risk of nonpharmacological and nonopioid pharmacological treatments.^5^ The National Institutes of Health (NIH)'s Helping to End Addiction Long-Term (HEAL) initiative identified research priorities to address the opioid crisis, highlighting the urgent need for enhanced pain management strategies, including advancing innovative, nonaddictive pain treatments and establishing effective pain management strategies for both acute and chronic pain conditions.^7^ Simply reducing the prescription (and supply) of opioid analgesics does not address the root of opioid use leading to addiction: the persistence of chronic pain. Both the CDC and NIH guidelines for chronic pain and opioid use explicitly state that nonpharmacological therapy or nonopioid pharmacological therapy is preferred for chronic pain.

Chronic pain has emerged as a treatment priority for PLH and is associated with psychological and functional morbidity as well as decreased retention in HIV primary care.^8^ Relatively half of the pain experienced by PLH is considered to be neuropathic and secondary to injury of the central or peripheral nervous systems from direct viral infection, infection with secondary pathogens, or side effects of medications.^9^ The prescribing of opioids in PLH is controversial for several reasons. Drug–drug interactions (DDIs) of varying degrees exist between all anti-retroviral therapy classes and select opioid analgesics, such as methadone, buprenorphine, meperidine, and fentanyl.^3,9,10^

In addition, current research indicates that PLH are over-represented among those with aberrant substance and opioid use disorders and chronic pain compared with the general public, thereby further increasing their risk of both addiction and adverse prescription opioid-related events.^3^

Abstinence from illicit substances is strongly correlated with viral suppression among PLH.^3^ Interestingly, even *reducing* frequency of opioid use without total abstinence was associated with viral suppression in this population.^11^ This further highlights the need for careful review of patient histories and consideration of appropriate mechanisms to treat chronic pain in an at-risk population.

Physical therapy (PT) is widely used for the mitigation and resolution of acute and chronic pain, and recent literature suggests that PT may play a valuable role in the care of PLH, including for chronic pain relief.^12–14^ In contrast to opioids, PT is widely regarded as a safe cost-effective low-risk treatment for chronic pain, and there is broad consensus that benefits of PT outweigh any potentially associated risks.^13,14^

This study aimed to identify which specific PT modalities and interventions are most effective in decreasing chronic pain and decreasing opioid use among PLH currently receiving chronic opioid therapy.

## Materials and Methods

This retrospective study investigated the effectiveness of a targeted PT intervention with the end-point of decreasing pain reports and opioid use among PLH enrolled at a large multidisciplinary AIDS clinic in the southeastern United States. The study was deemed exempt by the Institutional Review Board of the home institution, as it solely used de-identified patient information from retrospective chart review. The Ponce de Leon Center is located in metropolitan Atlanta, GA, and is one of the largest and most comprehensive HIV clinics in the United States serving the most vulnerable and at-risk populations in Atlanta. Metropolitan Atlanta was an ideal location for this study as the burden of HIV disease is high: Atlanta currently ranks fifth in the nation for total number of adults and adolescents living with HIV.^15^ The clinic has >6000 enrolled HIV-positive patients, with ∼90% of these patients identifying with under-represented minority groups. More than 70% of enrolled patients live below the federal poverty level; 42% are uninsured and 26% receive Medicaid. More than 70% of PLH who live in Atlanta reside within 2 miles of the Ponce Clinic, in an area recognized as a spatial clustering of the Atlanta HIV epidemic.^15^ Weekly PT has been available on-site at the clinic since 2014 and is one component of the multidisciplinary Palliative Care Program.

Inclusion criteria for this study included (1) HIV-positive adults age ≥18 years enrolled at the Ponce de Leon Center, (2) patients who received PT services at the Ponce de Leon Center between July 2014 and October 2017, (3) patients on chronic opioid therapy (>3 months), and (4) patients who had a noncancer chronic pain diagnosis (>3 months). Patients were seen for weekly PT sessions during the established timeframe by a single provider, with an average treatment period of 8 weeks. Although the duration of PT treatment may vary depending on diagnosis, severity, and indication, typical PT interventions for musculoskeletal pain last for an average of 8 weeks.^16^ De-identified charts were reviewed and selected based on the inclusion criteria. Subjects' pain scores were recorded at each PT visit, and compared between the initial PT evaluation (Time 1) and the PT discharge date (Time 2).

Pain scores were gathered from charts using the numerical rating scale, a standard 0–10 pain self-reporting scale that is the most widely implemented clinical scale for pain screening and has been shown to provide sufficient discriminative power for patients to describe their pain intensity.^17^ Pain medication prescription and dosage were also recorded. Patients were queried at each PT visit as to whether or not they were taking pain medications and whether or not these medications were prescribed or obtained somewhere other than from a medical provider. Analgesic prescriptions were verified through electronic charts where all prescriptions were recorded. Opioid use was measured by morphine milligram equivalents (MMEs), a standard measurement tool used to equate different types of opioids into one standard value allowing for comparisons and risk evaluations.

Patients received weekly 30-minute PT sessions ranging from 6 to 10 weeks in duration. PT interventions for chronic pain included home exercise programs, manual therapy, therapeutic exercise, education, soft tissue massage, menthol-based topical pain reliever, therapeutic taping (a rehabilitative taping technique that is used to decrease pain and inflammation as well as stabilize and support muscles and joints), stretching and transcutaneous electrical nerve stimulation (TENS). Current literature suggests that high-frequency TENS are as effective as an opioid-alternative for pain relief, because it specifically activates the mu-opioid receptors.^18,19^ All patients received self-management strategies and techniques at each visit, based upon their individual learning styles and needs.

Pre- and postintervention data collected included 0–10 pain score, pain medication prescriptions and usage, opioid use (MMEs), and PT evaluation data, including muscle strength, joint range of motion, and postural analysis. Each clinic PT visit collected pain scores and opioid use for that week, and recorded PT clinical interventions and patient tolerance/reaction to treatment. Pre- and postintervention data were compared for change in pain reports and opioid use/MMEs.

## Results

**FIG. 1.**
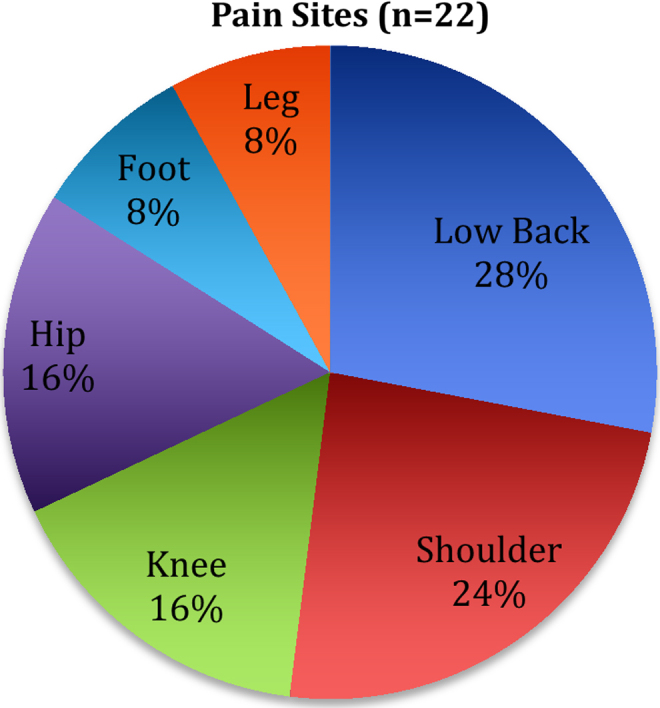
Pain sites (*n* = 22). Some participants had multiple pain sites in their referring diagnosis; therefore, total percentage is >100%.

All study participants (*n* = 22, 100%) had opioid analgesics prescribed to them. In addition to the opioids prescribed, 8% of patients had NSAIDS prescribed, 22% had acetaminophen prescribed, and 21% were prescribed neuropathic medication. All patients were uninsured and all lived below the federal poverty level. Of note, the PT program at this clinic treats exclusively uninsured patients, who are unable to receive PT services elsewhere due to financial barriers. Patients were all taking opioids for at least 3 months, with a range from 3 months to 7 years. Types of opioids taken were: codeine, hydrocodone, tramadol, morphine and oxycodone. Patients had a mean age of 54 (range: 23 to 78), a mean BMI of 36.8, 39.1% female, 60.9% male (no patients identified as transgender), 73.3% identified as African American, 22.2% as Caucasian, and 2.2% as Latino/Hispanic. The three most common referring pain diagnoses for subjects were low back pain (*n* = 7, 31.8%), shoulder pain (*n* = 6, 27.3%), knee pain and hip pain (each *n* = 4, 18.2%). Some participants had multiple pain sites in their referring diagnosis; therefore, total percentage is >100%. Figure 1 details chronic pain sites in order of prevalence.

A majority (*n* = 17, 77.3%) of subjects demonstrated a decrease in pain by the conclusion of the study period. The average pain scores for subjects at Time 1 and Time 2 were 7/10 and 1/10, respectively. Minimal clinically important difference (MCID) is a standard reporting mechanism that reflects clinical changes that are meaningful to a patient. The MCID for changes in musculoskeletal pain is a decrease in pain by one or more points (on a 0–10 scale). In this study, all patients who decreased pain reported over the MCID (a change of at least two points). Three (13.6%) subjects reported an increase in pain at various points for the study period, owing to reasons such as increased work or household activities, and 2 (9.1%) subjects had no change in pain.

**FIG. 2.**
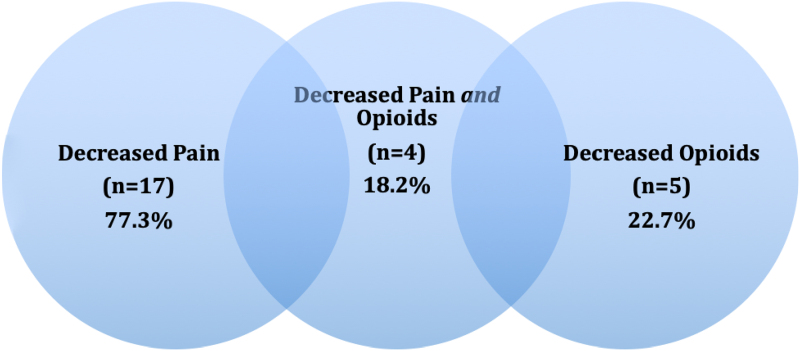
Postintervention pain and opioid outcomes (*n* = 22).

The average MMEs for subjects at Time 1 and Time 2 were 30.02 and 31.70, respectively. For those who decreased *both* pain and MMEs for the study period (*n* = 4, 18.2%), the following treatments were used most often: patient education on pain coping (*n* = 4, 100.0%), HEP (*n* = 4, 100.0%), massage (*n* = 3, 75.0%), stretches (*n* = 2, 50.0%), and therapeutic taping (*n* = 2, 50.0%). Of those who decreased in both pain score and opioids (*n* = 4, 18.2%), all received pain management education and home exercise programs. Figure 2 compares the percentages of participants who decreased pain, opioids/MMEs, and both.

Of the participants whose MMEs did not change or increased (*n* = 11, 50%), the majority (80%) of them still demonstrated a decrease in pain. Six (27.3%) subjects increased their opioid usage for the time period. Pain coping skills included encouraging patients to, at the initial onset of pain, perform three self-management techniques before taking an opioid: (1) perform their individualized home PT stretching/exercise routine; (2) put the TENS unit on the painful area for 20 min (clinically recommended time); (3) walk for 10–15 min. Pain coping techniques also included diaphragmatic/paced breathing with the following instructions: “Take a deep breath in slowly through your nose, letting your chest and lower belly expand. Breathe out slowly through your mouth with slightly pursed lips. Focus on the sound of your breath and the feeling of your chest and belly filling with air, then letting go.”

Descriptive statistics were used to analyze demographic data. A paired *t*-test was conducted to compare mean pain and MMEs and pain scores pre- and postintervention. For pain scores, there was a significant difference (<0.05) in the preintervention pain (M = 7.02, SD = 3.07) and postintervention pain scores (M = 1.39, SD = 3.07); *p* = 0.008, *t* = −9.021073, *p* = < 0.00001.

For pre- and postintervention MME values, there was a significant difference (<0.05) in the preintervention MMEs (M = 21.21, SD = 16.77) and postintervention MME scores (M = 6.1, SD = 3.07); *p* = 0.00032, *t* = −3.817245, *p* = < 0.00032.

## Discussion

Although current prevalence estimates of chronic pain among PLH remain high, this percentage may decrease in the next decade as we now have newer anti-retrovirals with increased tolerability and fewer side effects, have fewer DDIs with opioids, and that treatment with HIV is started sooner in HIV-diagnosed patients.^3^ Although we remain hopeful, it is essential to focus clinical and research efforts into nonopioid chronic pain management in this patient population. This will not only improve health-related quality of life, but could also potentially decrease aberrant opioid use.

A majority (*n* = 17, 77.3%) of patients in this sample demonstrated a decrease in pain after PT intervention. Of those who decreased in *both* pain score and opioid use (*n* = 4, 18.2%), all received pain self-management education/pain coping skills and home exercise programs. Notably, these are the only interventions that patients could do independently at home, rather than those that would need to be performed by the physical therapist. This may speak to subjects' empowerment of performing self-interventions at home to manage their pain independently. The pain self-management education utilized in this intervention could be potentially incorporated from patients' initial clinic visits for pain to improve overall pain and to reduce future opioid initiation and potential for addiction.

Of all the subjects who reported decreased or eliminated pain, only 22.7% concurrently decreased opioid use; some subjects had a decrease in pain scores, but their opioid usage remained stagnant or even increased. With these results, there is the potential for confounding of the outcome of decreased pain—specifically for those who increased their use of opioids during the study period. Of the participants whose opioid use/MMEs did not change or increase, the majority (80%) still demonstrated a decrease in pain after PT intervention. Although decreased pain reports are promising for the use of PT in chronic pain management in this population, our results highlight the need for focus on the complexity of opioid use and addiction. Decreasing or eliminating an individual's physiological pain is often not sufficient to concurrently decrease opioid use. Despite the fact that opioids are initially prescribed for such physiological, often chronic pain, the addiction and psychological components of both opioid dependence and chronic pain are key to successfully decreasing opioid use.

Amidst the multitude of challenges of the opioid public health crisis, the utilization of alternative/nonopioid methods to treat chronic pain must remain a priority. PLH are not immune to the opioid epidemic, and merit special consideration given higher incidences of both chronic pain and substance misuse.^3^ Recent literature suggests PLH have utilized opioid alternatives for chronic pain that cause less pharmacological burden, including complimentary alternative medicine (CAM).^20^ However, even some types of CAM can have undesirable side effects and even adverse interactions with antiretroviral medications.^20^

PT has been widely considered a safe effective nonpharmacological tool for chronic pain mitigation, and current literature promotes PT as part of the care team for PLH.^12–14^ It is important to acknowledge that solely exercise (aerobic and/or strength training) is not likely to be sufficient to meaningfully decrease chronic pain, but should be considered among the wide array of clinical skills included in physical therapists' training. A 2016 Cochrane review examined the effectiveness of different physical activity and exercise interventions in reducing pain severity and its impact on function, quality of life, and health care use (total of 381 studies and *n* = 37,143 participants).^21^ This review concluded that exercise alone was not associated with significant change (positive or negative) in self-reported pain scores.

Other studies have examined the effects of psychologically based pain management/pain coping skills in combination with orthopedic PT in chronic pain treatment.^22,23^ Results showed that after the interventions, participants who received both the psychologically informed pain coping and traditional PT reported better outcomes for disability, physical functioning, and overall physical health than those who received PT alone.^24^ The findings of these studies relate to the results of this study in that patient education surrounding pain coping, which includes a psychologically oriented patient-based approach, was a common denominator in participants who decreased both pain and opioid use. This could be a powerful training tool for physical therapists to better understand, empathize, and ultimately have more successful outcomes when treating patients with chronic pain, and highlights the fact that chronic pain and opioid use are multifactored issues that require a multidisciplinary approach.

The decrease in pain scores among subjects who received PT in this study and in other similar studies indicates that PT should be earnestly considered as a viable alternative to opioids when aiming to decrease chronic pain among PLH.

## Study Limitations

The small sample size (*n* = 22) is a primary limitation of the study, as it limits conclusions that can be drawn. In addition, because the study was not randomized we cannot be certain if patients who did not receive PT in the same timeframe would have achieved similar results in pain and MME levels. The subjects in this study were all patients at the same clinic. Future studies are needed in varying demographic and geographical areas with larger patient populations to account for variables in access to health care, community resources, and transportation.

Extenuating factors in patients' lives were not controlled for in the study due to the retrospective approach through chart review. For example, illicit drug use and self-medication with nonprescribed opioids may cause a decrease in pain regardless of the PT received. Owing to the retrospective design, these data were not available. In addition, the reasons why patients may have increased opioid use throughout the study period were not available. The patient population at this clinic has frequent exposure to psychosocial life stressors (trauma, unstable housing, and poverty) that may affect the care they have received to treat pain as well as the resources available to them to gain coping strategies. These significant life stressors were not recorded and, therefore, we were unable to correlate changes in pain/MMEs with psychosocial stressors. Chart review data did not include whether or not patients were currently receiving psychotherapy or other interventions to decrease opioid use. Future studies should include gathering this information, which may affect final outcomes of opioid use.

Finally, a limitation in the MME data is present. Opioid prescriptions and dosages were recorded from the physician prescription in the EMR. This may not accurately depict the individual's actual MMEs based on potential off-prescription opioid use, or patients' hesitation to be forthcoming regarding opioid use.

Whenever examining the relationship of chronic pain and opioid analgesics, the complexity of opioid use and addiction must be closely considered, with a focus on physiological, environmental, and psychosocial factors as well as access to mental health support. The potential for PT to effectively mitigate chronic pain in this patient population should ideally be considered within the context of a multidisciplinary team framework, which includes at the minimum primary HIV care, mental health services, and PT. As PLH continue to present clinically with both age- and HIV-related comorbidities, physical therapists can be a key member of the multidisciplinary team managing pain and improving the quality of life in this unique patient population.

## Abbreviations Used

CAM: complimentary alternative medicine
CDC: Centers for Disease Control and Prevention
DDIs: drug–drug interactions
HEAL: Helping to End Addiction Long-Term
MMEs: morphine milligram equivalents
NIH: National Institutes of Health
PLH: people living with HIV
PT: physical therapy
TENS: transcutaneous electrical nerve stimulation
